# MFPINC: prediction of plant ncRNAs based on multi-source feature fusion

**DOI:** 10.1186/s12864-024-10439-3

**Published:** 2024-05-30

**Authors:** Zhenjun Nie, Mengqing Gao, Xiu Jin, Yuan Rao, Xiaodan Zhang

**Affiliations:** 1 School of Information and Artificial Intelligence, Anhui Agricultural University, Hefei, 230036 China; 2 Key Laboratory of Agricultural Sensors, Ministry of Agriculture and Rural Affairs, Hefei, 230036 China

**Keywords:** Plants, ncRNA prediction, Fusion of deep feature and sequence feature

## Abstract

Non-coding RNAs (ncRNAs) are recognized as pivotal players in the regulation of essential physiological processes such as nutrient homeostasis, development, and stress responses in plants. Common methods for predicting ncRNAs are susceptible to significant effects of experimental conditions and computational methods, resulting in the need for significant investment of time and resources. Therefore, we constructed an ncRNA predictor(MFPINC), to predict potential ncRNA in plants which is based on the PINC tool proposed by our previous studies. Specifically, sequence features were carefully refined using variance thresholding and F-test methods, while deep features were extracted and feature fusion were performed by applying the GRU model. The comprehensive evaluation of multiple standard datasets shows that MFPINC not only achieves more comprehensive and accurate identification of gene sequences, but also significantly improves the expressive and generalization performance of the model, and MFPINC significantly outperforms the existing competing methods in ncRNA identification. In addition, it is worth mentioning that our tool can also be found on Github (https://github.com/Zhenj-Nie/MFPINC) the data and source code can also be downloaded for free.

## Introduction

RNA (ribonucleic acid) is a crucial molecule that plays diverse roles in cellular processes. Traditionally, RNA is primarily recognized as messenger RNA, acting as an intermediary messenger molecule in protein synthesis. However, recent research has unveiled a diverse array of non-coding RNA (ncRNA) [1, 2] molecules that do not encode proteins. Initially, it is dismissed as transcriptional “noise” or “junk” RNA [3, 4], but emerging evidence now suggests their critical regulatory functions across various biological processes, including gene expression, chromatin organization, and intracellular homeostasis. ncRNAs can be categorized into different classes based on their size and function. Well-explored ncRNA classes include transfer RNA (tRNA), ribosomal RNA (rRNA), small nuclear RNA (snRNA), small nucleolar RNA (snoRNA), and microRNA (miRNA) [5]. Of particular significance, miRNAs have garnered extensive attention due to their vital roles in post-transcriptional regulation. By binding to mRNA, they can either suppress translation or facilitate mRNA degradation. In addition to these ncRNA classes, long non-coding RNAs (lncRNAs) represent a versatile and functionally important group of ncRNAs. Typically exceeding 200 nucleotides in length, lncRNAs participate in the regulation of gene expression at multiple levels [6–8], including epigenetic modifications, transcriptional regulation, and post-transcriptional processing. They play crucial roles in development, cellular differentiation, and disease processes. In plants, non-coding RNAs exhibit manifold functions and regulatory actions. They actively contribute to various physiological processes such as nutrient balance, growth and development, response to biotic and abiotic stresses, as well as signal transduction [9]. For instance, certain ncRNAs have been implicated in the regulation of flowering time, leaf morphogenesis, root development, and response to environmental signals.

Predicting and identifying ncRNAs and other protein-coding RNAs(mRNAs) is a complex and challenging task [10]. Researchers have developed a variety of experimental and computational methods to unravel the ncRNA transcriptome. Experimental techniques such as northern hybridization, reverse transcription-polymerase chain reaction (RT-PCR), microarrays, and high-throughput RNA sequencing (RNA-seq) have been employed to detect and quantify ncRNAs [11]. On the other hand, computational methods utilize sequence homology, structure prediction, and machine learning algorithms to identify and annotate ncRNAs. While these methods have made significant progress in ncRNA prediction and identification, there is still a need for improved tools that offer higher accuracy while requiring less time and resources. In recent research, bioinformatics has emerged as a promising avenue for addressing biological challenges in this domain [12–14].

In 2007, Kong and colleagues introduced the Coding Potential Calculator (CPC) [15], representing a significant advancement in the field of bioinformatics. The design of CPC aimed to leverage crucial biological features to assist in predicting the potential coding functionality of sequences. These features encompass ORF (Open Reading Frame) quality, coverage, and completeness, which are seamlessly integrated into a Support Vector Machine (SVM) model for encoding potential biological attributes, although its performance is greatly influenced by sequence alignment. Over time, the field of bioinformatics has faced new challenges and opportunities. After 17 years, the release of CPC2 [16] further strengthened this tool. CPC2 not only achieved significant performance improvements compared to its predecessor CPC but also demonstrated enhanced speed and accuracy. As input for the SVM model, CPC2 introduced additional features, including ORF size and completeness, Fickett scores, and isoelectric points extracted from raw RNA sequences. This improvement rendered CPC2 a relatively neutral tool, better suited for studying the transcriptome of non-model organisms. Additionally, CPAT, introduced in 2013 by Wang et al., is another noteworthy tool. CPAT applies logistic regression modeling to classify ncRNAs and mRNAs based on features such as ORF size, coverage, Fickett score, and hexamer score [17]. Similarly, in 2013, Liang et al. proposed CNCI, which utilizes a distinct feature set relying on ANT features to classify ncRNAs and mRNAs, while utilizing the same SVM classifier as CPC2. Subsequently, in 2019, an updated version of CNCI [18], CNIT, was introduced, employing a more robust integrated machine model called XGBoost for classification. Another groundbreaking work was the introduction of CPPred [19] by Tong et al. in 2019. It is an SVM-based tool that utilizes a range of features, including ORF features similar to CPC2, along with isoelectric point, stability index, gravity tripeptide, hexameric score, CTD score, tripeptide and hexameric score, and CTD score, for classifying ncRNAs and coding RNAs.

Singh et al. have introduced a groundbreaking predictive tool for plant lncRNAs, which they have termed PLncPRO. This framework has utilized existing knowledge of both coding and non-coding transcriptomes and has employed a random forest algorithm to improve the accuracy of lncRNA detection [20]. Concurrently, Tatianne et al. have developed the RNAplonc approach, specifically designed for identifying lncRNAs within the plant kingdom. By conducting a thorough feature analysis of non-redundant plant transcriptome datasets within a machine learning framework, this method has successfully identified 16 key features that are instrumental in the categorization of lncRNAs [21]. However, it should be noted that these methodologies largely rely on plant lncRNA-specific datasets for model training, which may not fully capture the predictive and discriminative capabilities across the entire range of plant ncRNAs.Although numerous excellent tools have emerged for distinguishing between ncRNAs and coding RNAs, they still exhibit some limitations. One significant limitation is that these tools have primarily been trained and validated as models for vertebrates and mammals. Additionally, most of these tools have only been trained using the model plant *Arabidopsis thaliana*, limiting their applicability to non-model plants. It is worth noting that animal ncRNAs are predominantly synthesized by RNA polymerase II, while plant ncRNAs are mainly produced by RNA polymerases II, IV, and V [22]. Given the low-level expression and cross-species conservation characteristics of ncRNAs [23], these tools may not be sufficiently reliable when applied to the plant domain.

To address these issues, our lab released PINC in 2022 [24, 25], a tool based on the AutoML framework, which is able to extract 10 features that can effectively differentiate between non-coding and coding RNAs in plants. In comparison to the aforementioned four tools, PINC exhibits greater accuracy in identifying RNA sequences as non-coding RNAs. Nevertheless, for precise identification of non-coding RNAs, it is essential to carefully select high-quality features based on the distinctive structure of non-coding RNAs in plants. Achieving this requires a substantial amount of prior biological knowledge regarding these specific features.

The traditional feature extraction and feature selection methods in existing tools often focus on a single feature source, disregarding the interactions and synergies between different features. This limitation can lead to information loss and misinterpretation, consequently impacting subsequent data analysis and model construction. Therefore, in recent years, an increasing number of studies have explored feature fusion methods, which aim to effectively integrate features from different sources or at different levels [26]. The objective is to enhance the expressive and predictive capabilities of data. Feature fusion finds extensive applications in the field of bioinformatics, including gene expression data analysis, protein structure prediction, and drug target discovery [27, 28]. By leveraging feature fusion techniques, a few of researchers can harness the rich information contained in heterogeneous data from multiple sources, thereby improving the efficiency and accuracy of data analysis and model construction.

Therefore, this study proposes a feature fusion method to address the problem of ncRNA identification. Building upon the sequence features extracted by the PINC tool and validated through experiments conducted on the training set, we demonstrate that deep learning models can automatically extract plant-specific features from the raw sequence data. To further enhance the model’s performance, improve its generalization ability in identifying unknown gene sequences, provide richer information, and increase interpretability, our laboratory has developed a novel framework for non-coding RNA recognition. In this framework, we have considered the following aspects of our research: (1) a comparative analysis of different deep models for extracting depth features, (2) extraction of gene-related sequence features, and (3) fusion of depth features with sequence features.

The contributions of this paper are as follows:(1) we propose a deep feature extraction method based on a depth model; (2) we propose a method to perform fusion of deep features and sequence features; (3) in combination with the first two points, we develop a tool for ncRNA identification, and after comparing our tool with the PINC, ABLNCPP,  CPC2, CPAT, CNIT, and CPPred identification tools on 8 independent test sets to validate the performance of the tool, we found that our tool performed very well on these independent test sets. This indicates that our tool is equally a reliable method for identifying plant ncRNAs. In addition, in our web server, users can upload their own data for identification, which facilitates the study of plants that are currently receiving less attention.

## Result

### Comparison of depth models and acquisition of depth features

In this section, we engaged in a comparative analysis of seven widely-adopted deep learning algorithms within the natural language processing domain, specifically: RNN, Bi-RNN, GRU, Bi-GRU, LSTM, Bi-LSTM, and Transformer. To discern the most efficacious method, we employed one-hot and word-embedding encoding strategies throughout our experiments. In our experimental context, one-hot encoded a base utilizing a four-dimensional binary vector, while Word Embedding did so with a 50-dimensional vector. To uphold the comparison’s fairness across various encoding methods and models, we ensured consistency in experimental conditions. Our comparative results (referred to Table 1) revealed that the GRU, Bi-GRU, and Transformer models, when encoded with Word Embedding, outperformed their counterparts, registering significantly higher scores across all seven evaluation metrics relative to both the other four models encoded with Word Embedding and all models encoded with one-hot. Given the pronounced disparity between the two encoding methods in the context of our task, we chose Word Embedding as the preliminary encoding input method for the sequences. This decision initiatedthe enhancement of the GRU, Bi-GRU, and Transformer models.

**Table 1:** Performance of different deep learning models with different coding methods

CODING METHOD	MODEL	ACC(%)	MCC(%)	SE(%)	SPC(%)	PPV(%)	NPV(%)	F1(%)
ONE-HOT	GRU	86.08	66.08	91.59	80.34	82.93	90.16	87.04
	Bi-GRU	87.42	68.17	90.73	84.15	84.96	90.2	87.75
	LSTM	89.69	75	94.06	85.35	86.43	93.54	90.08
	Bi-LSTM	88.77	73.31	94.19	83.23	85.17	93.34	89.45
	RNN	85.46	62.17	88.06	82.78	84.06	87.05	86.01
	Bi-RNN	85.6	61.25	86.68	84.49	85.32	85.91	85.99
	Transformer	78.5	48.75	85.51	71.22	75.29	82.75	80.08
WORD EMBEDDING	GRU	**91.94**	**81.54**	**96.69**	**87.24**	**88.22**	**96.38**	**92.26**
	Bi-GRU	**91.42**	**81.12**	**97.24**	**85.61**	**87.06**	**96.89**	**91.38**
	LSTM	70.17	45.87	97	42.61	63.45	93.25	76.72
	Bi-LSTM	69.67	37.49	88.93	50.04	64.45	81.62	74.74
	RNN	87.1	68.43	91.85	82.33	83.93	90.86	87.72
	Bi-RNN	88.73	72.19	92.66	84.89	85.69	92.22	89.04
	Transformer	**92.35**	**82.16**	**96.76**	**87.82**	**89.11**	**96.33**	**92.78**

**Fig. 1:**
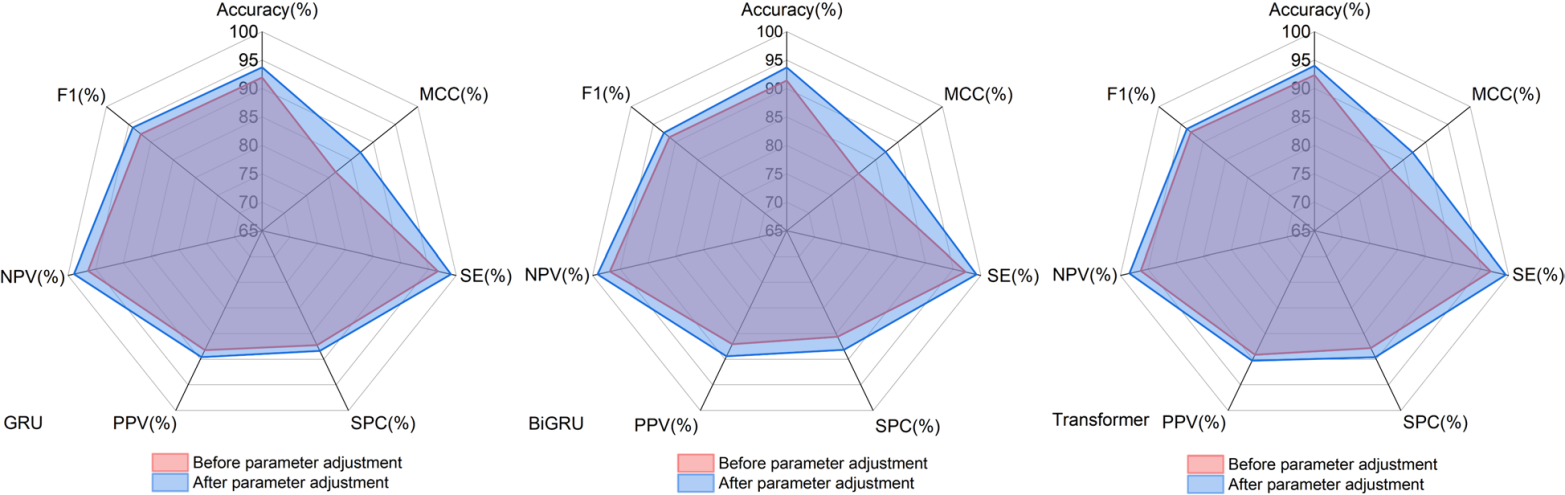
Performance of the model before and after parameter tuning

To optimize our model’s performance, we fine-tuned four distinct hyperparameters: learning rate, number of hidden units, batch size, and sequence length. For the learning rate, we evaluated the effects of four values: [0.1, 0.01, 0.001, 0.0001]. We tested three configurations for the number of hidden units: [20, 25, 30]. For batch size, we experimented with [8, 16, 32, 64], and for sequence length, we assessed the model’s response to inputs of 800, 100, 1200, and 1400 within our deep models,employing Word Embedding for encoding. This approach allowed us to gauge the impact of varying sequence lengths on the model’s recognition capabilities. The optimal hyperparameter configuration was determined to be: a learning rate of 0.001, 20 hidden units, a batch size of 16, and the optimal sequence length of the GRU and Bi-GRU models is 1200, and the optimal sequence length of the Transformer model is 1000.The detailed outcomes of this tuning process are illustrated below (Fig. 1).

**Fig. 2:**
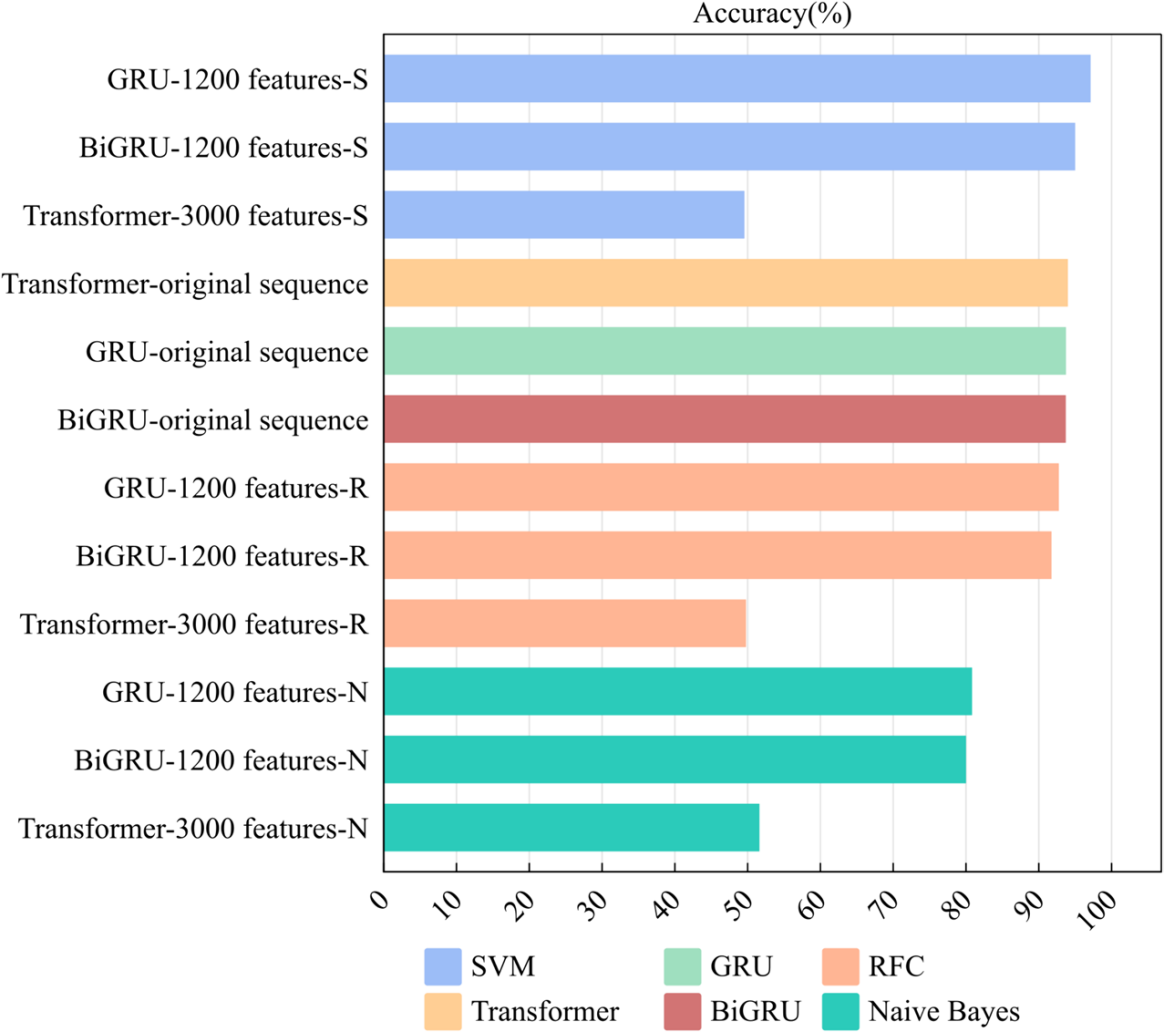
Comparison between the original depth model and machine learning models using three types of depth features(-S represents using the SVM model)

Considering the intricate structures and correlations typical of gene sequences, traditional feature extraction techniques may not be able to capture these complexities in capturing their nuance. To circumvent this limitation, we deployed three deep learning models—GRU, Bi-GRU, and Transformer—to autonomously discern potential features from these sequences. Upon optimizing these models, we extracted 1,200 deep features from GRU, 1,200 from Bi-GRU, and 3,000 from Transformer, calibrating the filter dimensions for each model to [3, 5]. Deep learning models, with their multi-layered architecture, excelled in automatically recognizing and portraying the high-dimensional features inherent in gene sequences, thereby enriching the representation of pivotal genetic data. To validate the efficacy of these extracted deep features, we compared the prediction effect of these three deep features with the original sequence on the deep learning model in three common machine learning models as a way to verify whether the effect of deep features was feasible, and the results were shown below (Fig. 2).

From the bar charts, the performance differences of various deep features across machine learning models were evident. Notably, barring the 3,000 features extracted from the Transformer, both GRU and Bi-GRU exhibited exceptional performance in SVM and RFC. The discrepancies between them and the initial deep learning model were minimal, spanning just 1% and 2%, respectively. These findings underscored the efficacy of our approach in harnessing deep learning models for feature extraction from gene sequences and emphasize the pivotal role that deep feature extraction plays in RNA sequence recognition. Deep learning models excel in autonomously discerning abstract, high-dimensional representations that are essential to encapsulate the intricate compositional and structural nuances within RNA sequences. This capability obviates the need for labor-intensive manual feature engineering and amplifies the efficiency in capitalizing on the data’s inherent richness. Furthermore, these models adeptly capture both local and global dependencies within RNA sequences via multi-layered representation learning. This facilitates a nuanced understanding of the interplay among the bases and the overarching sequence architecture, resulting in a heightened precision in predicting RNA sequence attributes.

Furthermore, we observed that the 3,000 deep features derived from the Transformer achieved an impressive 94% accuracy within the original deep model. However, their performance in random forests was lackluster. This disparity suggested that while the Transformer model boasted potent expressivity and generalization capabilities, it demanded ample data and computational resources for effective training and optimization. Drawing from this, it’s evident that Deep Learning models excel in gleaning deep features from gene sequences, with particularly favorable outcomes when applied to random forests.

### Comparison of sequence features and depth features

For feature extraction, our emphasis was on relevance and discriminative power. We employed variance thresholding during our experiments for feature selection. In particular, our selection encompassed 91 features, inclusive of k-mers = [1,2,3] and CDs correlation, alongside 45 features that surpassed the mean threshold. We further incorporated a hybrid method combining the f-test with variance thresholding using the PINC tool. This allowed us to pinpoint 10 salient features, namely: GC content, Score, cdsStop, cdsSize, T, C, GT, GC, ACG, and TAT frequencies.

**Fig. 3:**
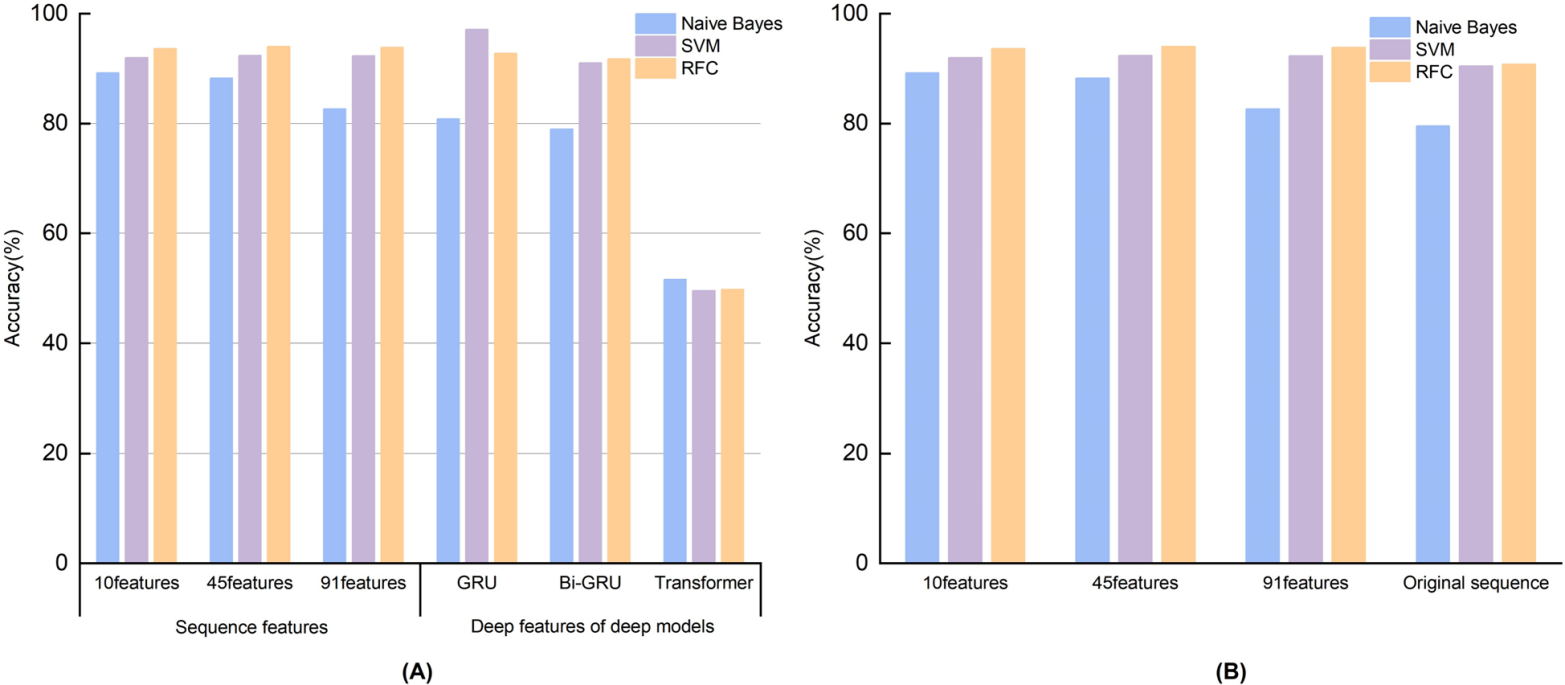
**A** Comparison of three sequence features and three deep features in different machine learning models; (**B**) Comparison of three sequence features and the original sequence in different machine learning models

Post-feature extraction, we fed the unfiltered 91 features and the post-filtered 45 and 10 features into three machine learning algorithms: Naive Bayes, SVM, and RFC. To ascertain the significance of these sequence characteristics in the context of gene sequence modeling, we conducted a comparative analysis of their predictive efficacy against the deep features derived from the aforementioned trio of deep learning models, utilizing a designated validation dataset. Concurrently, we also compared these three sequence features with The original sequences without extracted features were compared. Detailed results are shown below (Fig. 3).

Upon thorough examination of the data and rigorous evaluation of the models, Fig. 3(A) provides a clear depiction of the accuracy performance associated with manually curated sequence characteristics across various machine learning frameworks. Notably, the incorporation of these features into the RFC model exhibits performance that is competitive with the two superior deep learning architectures, namely the GRU and the Bi-GRU. Specifically, within the RFC model, the accuracy achieved with the utilization of 10, 45, and 91 sequence features was 93.63%, 94.02%, and 93.83%, respectively. These figures are marginally lower by 0.0088 and 0.0127 when compared to the accuracy of the GRU’s deep features within the same model, highlighting the robustness of the manually extracted features.

Furthermore, Fig. 3(B) presents a comparative analysis revealing that the utilization of extracted sequence features markedly enhances the predictive accuracy of the model when contrasted with the original sequences devoid of feature extraction. Within the Random Forest Classifier (RFC) model, which demonstrated optimal performance, the incorporation of the three distinct feature sets resulted in a significant increase in accuracy by 0.0282, 0.0321, and 0.0302 percentage points, respectively, over the accuracy achieved with the original sequence. These findings substantiate the efficacy of the sequence feature extraction technique employed in our study.

It is clear from the figure that the manually extracted sequence features outperform the deep features of the deep model on each machine learning model of the validation set. It’s pivotal to note that our validation and test sets were analogous in terms of data distribution, noise, and outlier presence. Such a consistency led us to surmise that machine learning models might possess enhanced generalization capabilities, aptly fitting smaller datasets. For tasks involving sequence recognition, the integration of manually curated features appears to bolster model efficacy. In some instances, these sequence-specific features are likely more pertinent and discerning than features learned autonomously, potentially accounting for the observed superior performance of the machine learning model on the test set.

From the presented figure, it’s evident that employing the extracted feature sequences as inputs into the machine-learning model yielded remarkable accuracy rates. Notably, the RFC stood out when these features were utilized, showing a significant advantage over the other machine learning models and matching the accuracy of the deep learning model. One plausible explanation was the inherent design of the RFC: an ensemble learning model that amalgamates the predictions from multiple decision trees through a voting mechanism. Such an architecture enriches the model’s complexity, enabling it to discern intricate patterns within the data. In contrast, both Naive Bayes and Support Vector Machines (SVMs) predicate their predictions on singular hypotheses or decision boundaries. Furthermore, RFC possesses intrinsic mechanisms to counteract overfitting by constructing each decision tree from a random subset of features and samples. This inherent randomness promotes model diversity, curtailing overfitting risks. Conversely, both Naive Bayes and SVMs might necessitate supplementary parameter tuning or regularization to mitigate overfitting. These findings accentuated the pivotal role of manually extracted features in sequence prediction tasks.

To ensure the RFC’s resilience against overfitting and its commendable generalization capability, we evaluated three distinct feature sets across three machine learning models—plain Bayes, SVM, and RFC—on eight independent test sets. The subsequent table revealed that the RFC consistently surpasses its counterparts on the validation set, irrespective of the feature dataset employed (Table 2). Such results underscored the robust generalization aptitude of our chosen RFC.

**Table 2:** Comparison of different sequence feature numbers in test sets in simple bayes, random forest, and support vector machines

Species	10 Sequence features			45 Sequence features			91 Sequence features		
	Naive Bayes	SVM	RFC	Naive Bayes	SVM	RFC	Naive Bayes	SVM	RFC
Cicer arietinum	90.04	92.95	**94.7**	87.82	93	**94.97**	77.93	93	**94.77**
Gossypium darwinii	89.5	91.91	**93.51**	91.02	92.02	**93.64**	87.57	92.02	**93.55**
Lactuca sativa	88.67	91.31	**93.27**	85.96	91.46	**93.26**	74.51	91.46	**93.26**
Manihot esculenta	87.32	89.84	**91.24**	89.28	89.88	**92.66**	88.22	89.88	**91.13**
Musa acuminata	89.96	91.95	**92.94**	89.3	92.07	**93.45**	84.72	92.07	**93.45**
Nymphaea colorata	91.72	94.55	**95.75**	92.25	94.63	**95.87**	90.21	94.63	**95.87**
Sorghum bicolor	89.32	91.36	**93.2**	93.39	91.44	**93.56**	**93.61**	91.44	93.22
Zea mays	89.85	92.41	**94.24**	93.75	92.01	**94.71**	92.79	92.03	**94.24**

### Performance comparison after fusion of features

**Fig. 4:**
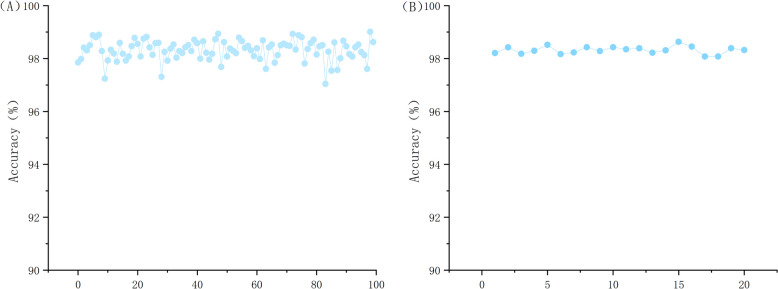
**A** Graph showing the accuracy of 100 experiments; (**B**) Graph showing the average accuracy of every 5th experiment out of 100 experiments

Once the machine learning model was finalized, we conducted multiple iterative experiments using the 91 sequence features mentioned previously, in conjunction with 1200 GRU deep features, within the selected RFC framework. As depicted in Fig. 4(A), across 100 trials, our accuracy peaked at 98.72% and bottomed out at 97.52%, with the majority of results falling between 97.8% and 98.7% (Fig. 4). This narrow range underscored the minimal variance in our outcomes. To offer a clearer perspective on the consistency, we averaged the accuracy over every five trials and represented this data as a curve in Fig. 4(B). This visualization showcases even smoother fluctuations, reinforcing the robustness of our results. These findings suggested that the integration of sequence and deep features, as orchestrated in our approach, leverages the strengths of both, culminating in enhanced model performance. Our adopted feature fusion methodology demonstrates impressive stability and precision in the domain of gene sequence classification. Furthermore, this successful marriage of feature types furnishes an efficacious and dependable strategy to tackle gene sequence classification challenges. Our tests also affirmed that repetitive experimentation, followed by computing mean accuracy, was a reliable tactic to diminish the potential sway of random variables, ensuring more trustworthy evaluations.

**Fig. 5:**
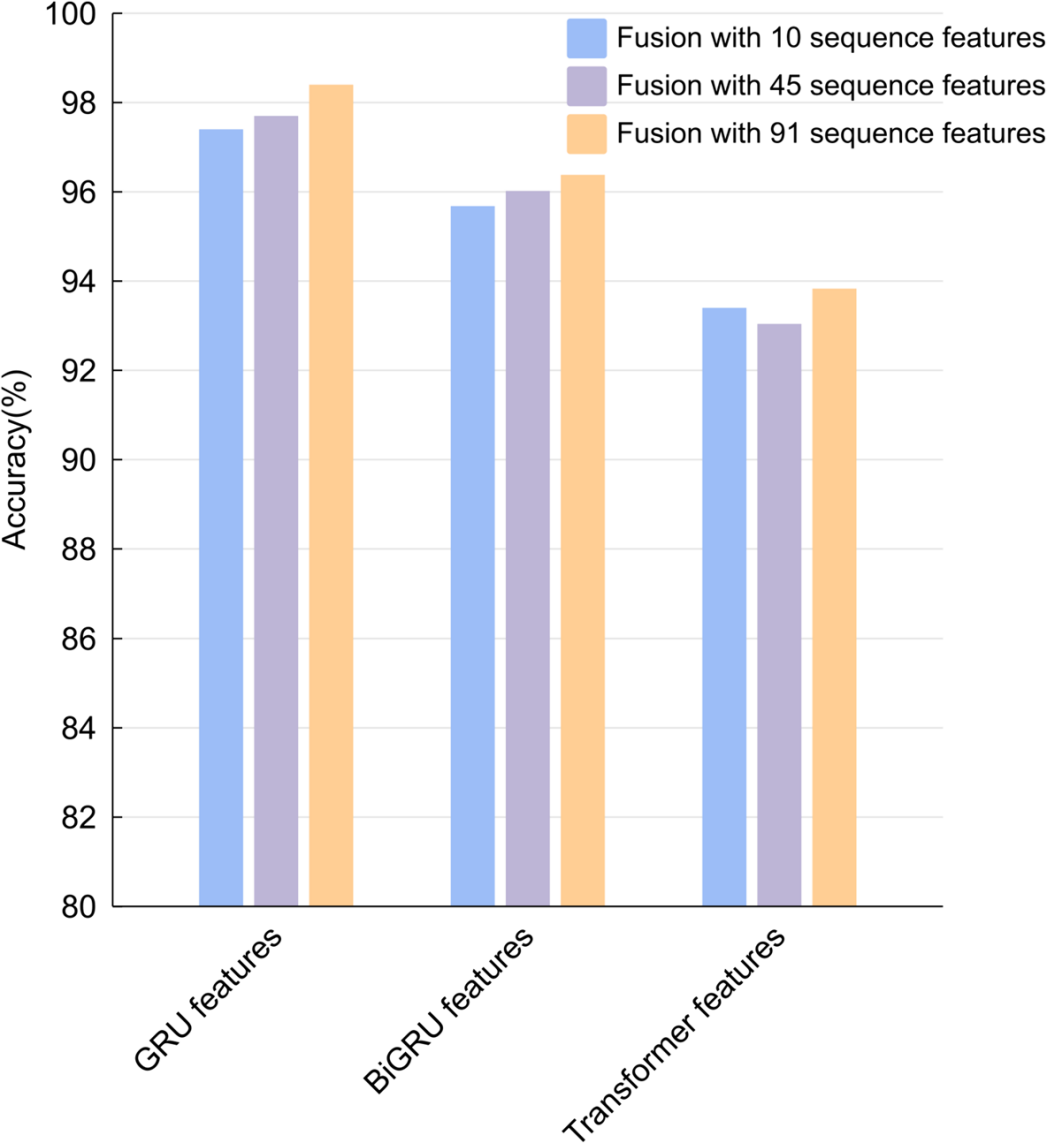
Comparison of fusing multiple quantities of depth features with different sequence features

To substantiate the representative capacity of RNA sequences, we utilized a stratified selection of previously delineated sequence features, specifically in quantities of 10, 45, and 91, and amalgamated them with the deep features derived from the superior-performing GRU, Bi-GRU, and Transformer models. In our analysis, we systematically extracted a distinct set of deep features from each of the three aforementioned deep learning models: 1200 features from the GRU model, an equivalent number from the Bi-GRU model, and a more extensive set of 3000 features from the Transformer model. As depicted in Fig. 5, our findings indicate that across various feature combinations, the confluence of features extracted by any of the models with the 91 sequence features yields an accuracy peak of 98.4%. This peak is notably superior to the accuracies achieved with the fusion of either 10 or 45 sequence features, which were found to be 0.01 and 0.007 percentage points lower, respectively. Given the elevated accuracy observed in the validation set post-feature fusion, and considering that the fusion of 10 and 45 features did not surpass the threshold of 98%, we have opted to employ all 91 extracted sequence features for subsequent experimentation.

Simultaneously, Fig. 5 elucidates that the fusion of 91 sequence features with GRU deep features elevated the model’s accuracy to 98.4%. This outcome surpasses the accuracies achieved by the amalgamation with the Bi-GRU and Transformer models by 0.0202 and 0.0432 percentage points, respectively. Observations indicate that the integration of features predominantly enhances the accuracy of the validation set; however, the Bi-GRU and Transformer models exhibit a significant disparity in accuracy when compared with the GRU model. Consequently, in subsequent experiments, we have opted to exclusively integrate all 91 extracted sequence features with the GRU deep learning model as the input feature set, thereby facilitating a more in-depth assessment and evaluation of the model’s performance.

**Fig. 6:**
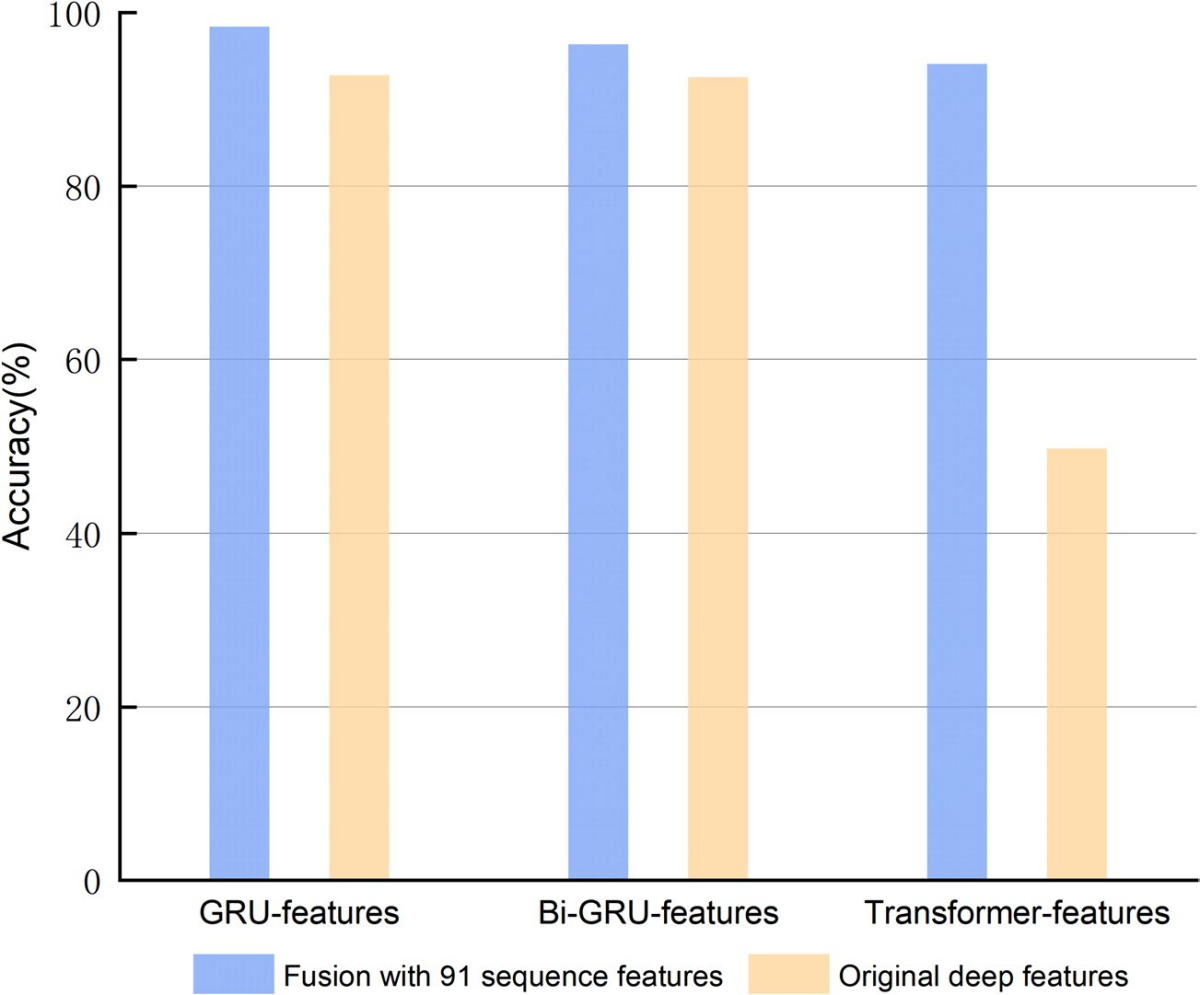
Comparison of deep features and 91 sequence features before and after fusion in RFC models

Subsequently, we employed the proficient RFC models, established from the preceding sections, within the validation set to evaluate the predictive efficacy across diverse feature categories. This encompasses comparisons of 91 sequence features, each synergistically fused with deep features extracted from three disparate models, as well as juxtapositions with three sets of unfused features. The accuracy of the six models within the validation set was illustrated via a bar chart, depicted in the following figure (Fig. 6).

As depicted in the preceding figure, it was evident that employing 91 sequence features and subsequently fusing them with deep features derived from three distinct deep learning architectures significantly enhances accuracy. This enhancement attested to the efficacy of our fusion technique. We posited that combining sequence and deep features harnessed the inherent complementarity and diversity between the two, thereby elevating both the representational capability and classification outcomes for RNA sequences. Furthermore, upon examination, we discerned varying performances of the RFC model under different quantities of sequence features post-fusion with GRU, Bi-GRU, or Transformer deep features. Notably, the RFC model with GRU deep features consistently outperformed others across all scenarios. This supremacy was succeeded by the Bi-GRU fusion, with the Transformer fusion trailing behind. Such distinctions can potentially be ascribed to the GRU’s cleaner and efficacious gating mechanism, enabling it to glean more stable and robust insights from RNA sequences.

**Fig. 7:**
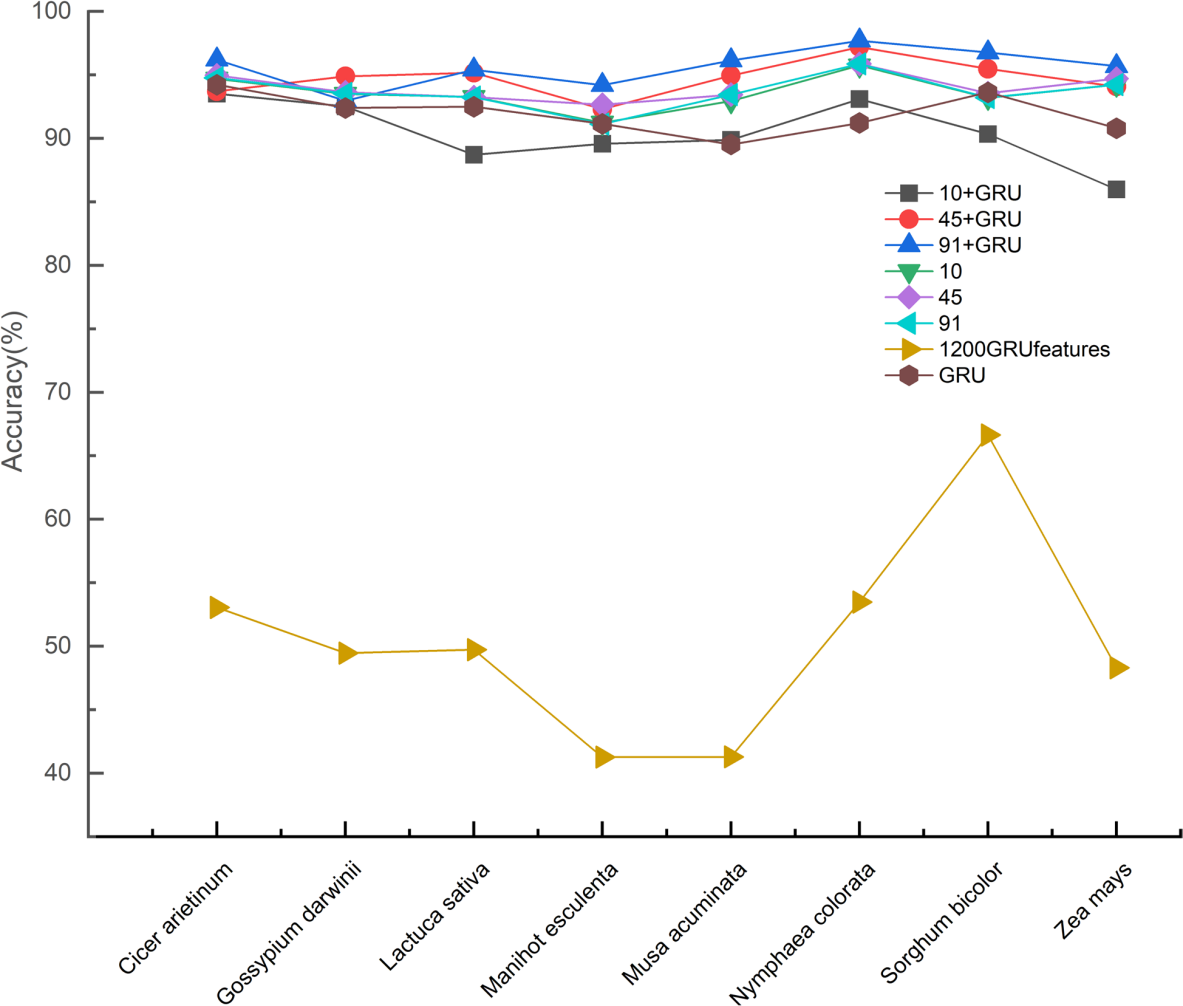
Comparison of recognition accuracy before and after feature fusion

To assess the effectiveness of our feature fusion approach in cross-species predictive recognition and conduct a comparative study, we conducted performance evaluations on eight independent test sets using various models. We compared the following scenarios: fusion of manually extracted 10, 45, and 91 species features with 1200 GRU depth features; utilization of solely unfused 10, 45, and 91 sequence features; utilization of only 1200 GRU depth features; and employment of the GRU depth model. The experimental results were presented in the figure below (Fig. 7).

It was evident from the figure that the feature fusion method demonstrated the highest accuracy on all test sets, significantly enhancing the classification performance compared to individual sequences or deep features. This signified the successful integration of sequence features and deep features in our feature fusion method, resulting in improved model generalization and robustness. Additionally, we also observed that the GRU deep model performed well on most test sets, ranking second after the feature fusion approach and occasionally surpassing it. This highlighted the ability of the GRU deep model to autonomously learn crucial information from gene sequences, eliminating the need for manually engineered features. In contrast, the performance of a single sequence or deep features varied inconsistently across different test sets, sometimes yielding better results and sometimes worse. This inconsistency could be attributed to the limited ability of individual sequences or deep features to capture the complexity and diversity of gene sequences, leading to model ineffectiveness or underfitting on specific species. In conclusion, our research results validate the effectiveness and reliability of the feature fusion method in cross-species predictive identification. The successful application of feature fusion provides a viable approach to address gene sequence classification challenges.

### Comparison to other tools

**Fig. 8:**
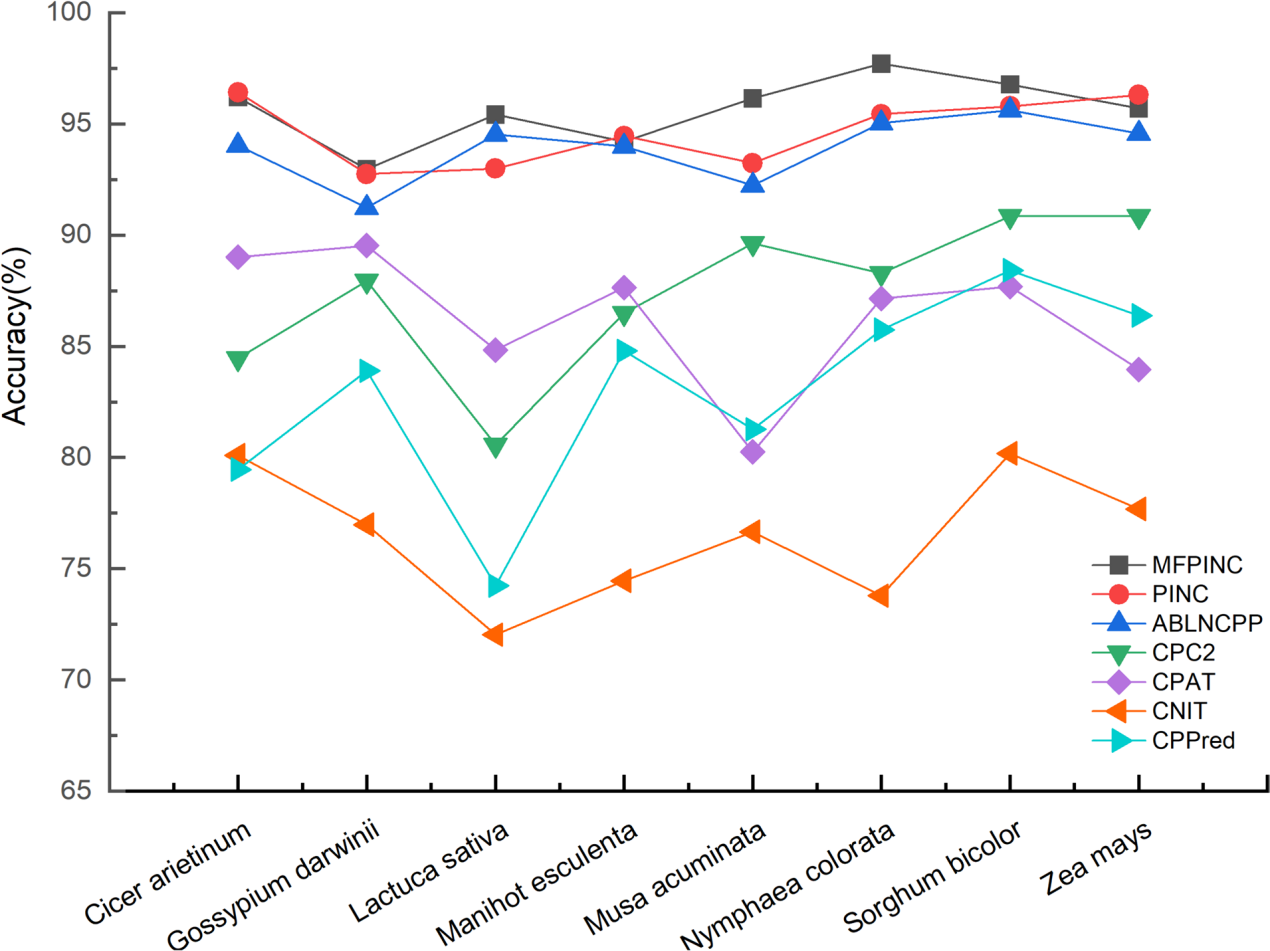
Compare the recognition accuracy of the other six tools in eight independent test sets

To evaluate the accuracy of our method in the identification of non-coding RNA and coding RNA, we conducted a comparative analysis with five tools: PINC, ABLNCPP, CPC2, CPAT, CNIT, and CPPred. We gathered datasets from eight plant species across four databases, namely GreeNC, CANTATA, RNAcentral, and Phytozome, and subjected them to analysis using these six different tools. The results depicted in the figure below clearly illustrate that our tool exhibits a notably high prediction accuracy across all eight species and performs with the most accuracy in the predictive identification of five of them (Fig. 8). In contrast to the other five tools, CPPred exhibits higher fluctuations, indicating a relatively weaker generalization capability. On the other hand, the remaining four tools demonstrate a level of stability, with PINC, in particular, slightly outperforming our method in the prediction of certain species.To further evaluate the performance of these tools across different species, we assessed them using eight evaluation metrics: accuracy (ACC), F1 score, AUC, MCC, NPV, PPV, sensitivity (SE), and specificity (SPC) on eight independent test sets. The results are presented below (Table 3). These findings demonstrated that our tool performs well in distinguishing non-coding RNAs from coding RNAs. *Musa acuminata* (banana) consistently outperformed the other tools in all evaluated metrics and test sets. It displayed higher values for SE, ACC, F1, NPV, and MCC, indicating a lower probability of missed predictions. Therefore, our tool is regarded as the top choice for non-coding RNA identification.

**Table 3:** Comparison of assessment indicators for eight plant species using seven tools

Species	Tool	ACC (%)	F1 (%)	AUC (%)	MCC (%)	NPV (%)	PPV (%)	SE (%)	SPC (%)
*Cicer arietinum*	MFPINC	96.2	96.15	96.27	**92.48**	**98.22**	94.21	98.16	**94.38**
	PINC	**96.42**	**97.2**	95.61	92.34	97.72	**95.7**	**98.76**	92.46
	ABLNCPP	94.05	93.73	94.62	88.21	92.04	94.69	96.74	93.64
	CPC2	84.45	83.04	**96.5**	69.92	79.42	91.5	76.01	92.91
	CPAT	89.01	89.05	96.26	78.02	89.18	88.84	89.27	88.75
	CNIT	80.1	76.81	94.36	62.99	73.23	92.54	65.65	94.67
	CPPred	79.46	77.65	89.72	59.75	75.24	85.32	71.24	87.7
*Gossypium darwinii*	MFPINC	**92.96**	**93.19**	92.97	**86.13**	96.16	**90.17**	96.43	89.52
	PINC	92.74	93.16	92.72	86.08	**98.41**	88.29	**98.61**	86.84
	ABLNCPP	91.23	92.36	93.05	84.66	92.58	86.43	94.71	88.64
	CPC2	87.94	87.6	**95.23**	75.99	86	90.09	85.25	90.62
	CPAT	89.53	90.07	93.65	79.54	94.42	85.61	95.03	84.02
	CNIT	76.98	73.31	90.67	56.13	71.16	87.21	63.23	**90.73**
	CPPred	83.89	82.36	91.66	67.59	83.8	84	80.78	86.59
*Lactuca sativa*	MFPINC	**95.41**	**95.37**	**95.41**	**90.81**	95.31	**95.5**	95.23	**95.58**
	PINC	92.99	93.47	92.9	86.54	**98.6**	88.68	**98.8**	87
	ABLNCPP	94.52	94.12	93.63	89.39	91.03	93.52	89.76	94.96
	CPC2	80.56	78.3	93.56	62.49	75.3	88.58	70.14	90.96
	CPAT	84.84	85.03	92	69.79	86.9	82.94	87.24	82.5
	CNIT	72.03	65.08	89.79	48.26	65.59	87.09	51.95	92.24
	CPPred	74.23	71.42	84.12	49.42	70.24	80.16	64.39	84.06
*Manihot esculenta*	MFPINC	94.2	94.23	**94.43**	88.89	99.21	89.71	99.22	**89.63**
	PINC	**94.45**	**95.4**	93.47	**88.99**	**99.72**	**91.36**	**99.82**	87.12
	ABLNCPP	93.95	93.99	92.85	89.89	93.91	90.99	96.47	86.32
	CPC2	86.48	86.66	92.15	72.99	87.48	85.53	87.82	85.14
	CPAT	87.64	88.33	91.13	75.81	92.68	83.66	93.55	81.73
	CNIT	74.46	71.06	91.3	50.33	69.82	81.94	62.73	86.18
	CPPred	84.79	85.19	88.97	69.68	86.78	83	87.5	80.08
*Musa acuminata*	MFPINC	**96.14**	**96.28**	**96.14**	**92.53**	**99.79**	**93**	**99.81**	**92.47**
	PINC	93.23	93.61	93.22	87.08	99.11	88.61	99.22	87.22
	ABLNCPP	92.23	92.24	95.35	84.5	90.77	92.68	91.03	93.5
	CPC2	89.62	90.6	94.71	79.02	87.83	91.09	90.12	88.99
	CPAT	80.25	78.41	91.84	61.42	75.79	86.54	71.69	88.82
	CNIT	76.66	73.63	90.14	54.85	71.6	84.77	65.08	88.28
	CPPred	81.27	80.33	89.24	62.84	78.48	84.64	76.44	86.1
*Nymphaea colorata*	MFPINC	**97.7**	**97.68**	**97.7**	**95.4**	97.49	**97.91**	97.45	**97.65**
	PINC	95.44	95.66	95.38	91.08	**98.7**	92.69	**98.82**	91.94
	ABLNCPP	95.03	95.58	93.06	90.38	96.61	91.54	97.32	89.24
	CPC2	88.28	87.57	97.08	77.03	84.45	93.06	82.69	93.85
	CPAT	87.14	86.57	95.1	74.56	84.24	90.59	82.9	91.39
	CNIT	73.79	67.81	92.24	51.27	67.34	87.88	55.21	92.38
	CPPred	85.74	85.62	91.56	71.49	85.14	86.36	84.89	86.59
*Sorghum bicolor*	MFPINC	**96.77**	96.7	**96.76**	**93.54**	96.42	**97.15**	96.25	**97.27**
	PINC	95.79	96.92	93.79	90.69	**99.79**	94.11	**99.9**	87.69
	ABLNCPP	95.61	96.08	95.79	91.47	98.62	92.46	97.32	90.51
	CPC2	90.85	91.16	96.42	81.91	93.95	88.16	94.38	87.32
	CPAT	87.68	87.55	95.71	75.38	86.93	88.46	86.65	88.71
	CNIT	80.19	79.1	92.89	60.71	77.4	83.63	75.04	85.34
	CPPred	88.42	88.8	94.28	77.02	91.21	85.98	91.81	85.04
*Zea mays*	MFPINC	95.69	95.46	95.84	91.46	98.09	93.15	97.88	**93.8**
	PINC	**96.3**	**97.16**	95.04	**92.12**	**99.45**	**94.74**	**99.71**	90.38
	ABLNCPP	94.66	94.75	94.38	89.34	95.61	93.77	95.75	93.55
	CPC2	90.85	90.84	**96.63**	81.7	90.82	90.87	90.81	90.88
	CPAT	83.96	82.64	95.07	68.67	79.63	89.82	76.52	91.37
	CNIT	77.69	74.49	92.5	57.15	72.24	86.78	65.24	90.1
	CPPred	86.38	86.16	93.05	72.8	85.29	87.54	84.83	87.92

In comparison with the current state-of-the-art ncRNA prediction tool ABLNCPP, our proposed MFPINC model demonstrates marked superiority across several pivotal evaluation metrics. Specifically, the MFPINC model has attained enhanced accuracy (ACC) and F1 score on eight distinct test datasets, underscoring its adeptness at achieving an optimal equilibrium between precision and recall. Furthermore, the MFPINC model exhibited superior performance across all datasets except for the Gossypium Darwinii dataset, particularly on the AUC index—a metric that quantifies classification efficacy. This observation further corroborates the model’s heightened competence in discerning diverse categories of ncRNA.Furthermore, among these eight plant datasets, our tool exhibited superior performance in five of them, surpassing the other five tools across at least five evaluation metrics, including accuracy (Accuracy). In the remaining three datasets, our tool’s predictive accuracy was comparable to that of PINC, yet notably outperformed the other four tools.

**Fig. 9:**
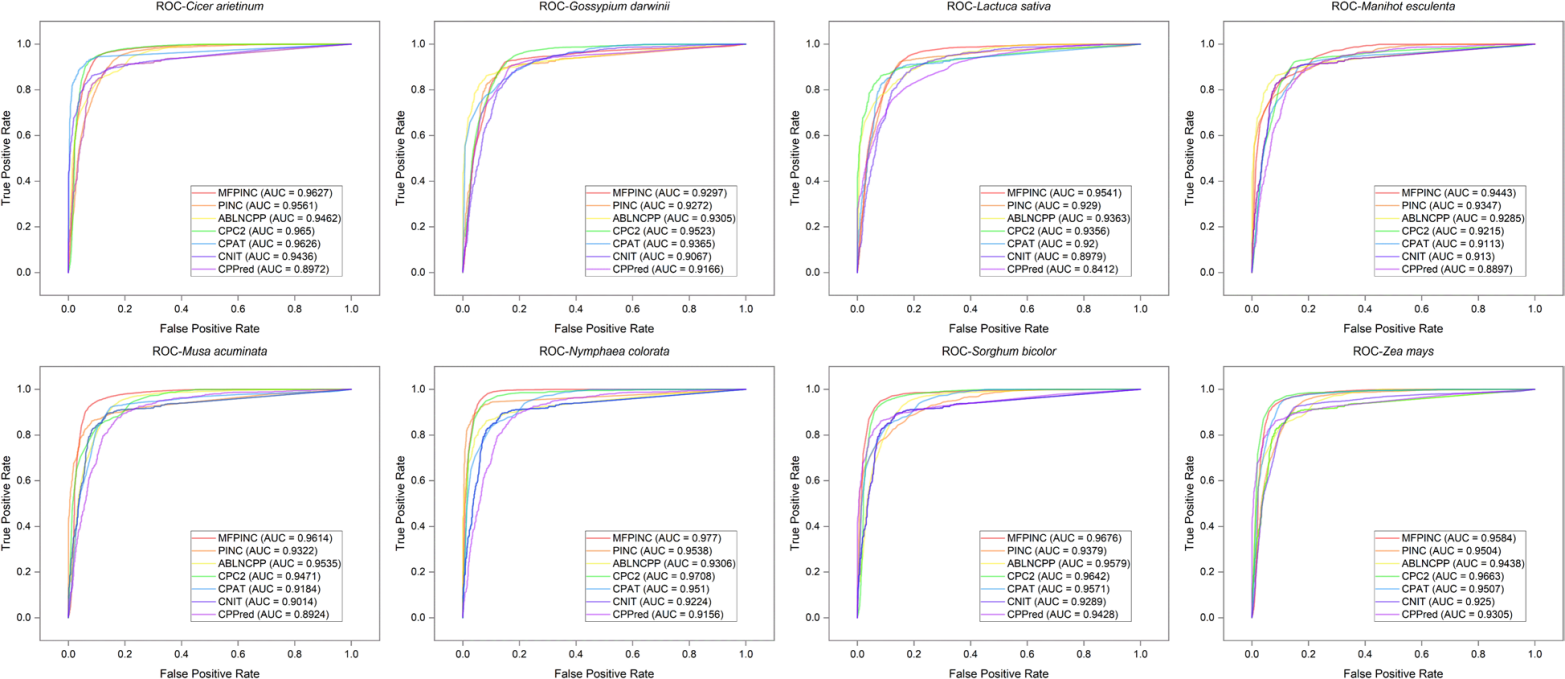
The ROC curve of all 7 tools on the 8 species datasets

Additionally, we constructed ROC curves, as depicted in Fig. 9, to assess the efficacy of various models in discriminating between ncRNAs and coding RNAs. The ROC curve corresponding to the MFPINC model demonstrated commendable performance, rapidly attaining a high true positive rate (approaching 1.0) while concurrently sustaining a low false positive rate across a spectrum of plant species. Notably, the MFPINC model achieved the highest area under the curve (AUC) across multiple species, signifying an optimal balance between the True Positive Rate (TPR) and the False Positive Rate (FPR) across all evaluated thresholds. Moreover, the AUC value for the MFPINC model surpassed 0.95 in several plant species, with an AUC value nearing 1.0 indicative of the model’s exceptional discriminative capacity. This implies that the model is highly proficient in accurately classifying both positive and negative samples with a high degree of probability. This statistical metric further substantiates the model’s elevated accuracy and reliability in the identification of ncRNAs and coding RNAs.

Consequently, within this set of six tools, our method proved to be the most effective for identifying noncoding RNAs in these eight plant species. This underscores the robust generalization capacity of our tool for predicting and identifying plant RNA sequences, which holds particular significance for non-model plant species.

### Statistical significance of methods performance

In recent years, both the z-test and t-test have gained widespread popularity as statistical methods for comparing significant differences between methods. Specifically, the z-test is well-suited for experimental methods with larger sample sizes. In the context of this study, the datasets for the eight species under examination encompass a substantial number of ncRNAs and coding RNAs. Therefore, the z-test is employed to assess whether the differences observed between the MFPINC method and the six methods, namely PINC, ABLNCPP, CPC2, CPAT, CNIT, and APPred, are statistically significant. To assess the significance of the observed differences between the methods, we conducted one-tailed tests within the z-test framework and calculated the corresponding *p*-values. The paper makes use of the SciPy library in Python to calculate *p*-values. SciPy is a robust numerical computing library that offers a wide range of functions specifically designed for statistical analysis, including the capability to compute *p*-values. To perform the one-tailed tests, we calculated the z-values based on Eq. (1)

1 $$Z\;=\;\frac{f_{12}\;-\;f_{21}}{\sqrt{f_{12}+f_{21}}}$$

The variable ${f}_{12}$ denotes the instances of ncRNA correctly classified by the first method yet misclassified by the second method, whereas ${f}_{21}$ signifies the instances of ncRNA incorrectly classified by the first method but correctly by the second. A calculated z-value surpassing 1.64 (with P < 0.1) is indicative of a statistically significant difference at a 90% confidence level in the accuracy of ncRNA classification between the two methods. According to the findings presented in Table 4, when compared to the MFPINC method, both the PINC and ABLNCPP methods demonstrated significant differences in classification accuracy solely within the datasets corresponding to *Gossypium darwinii* and *Nymphaea colorata* out of the eight independent test sets evaluated. The remaining six datasets demonstrated less pronounced statistical variances. Conversely, the CPC2, CPAT, CNIT, and CPPred methods displayed substantial differences in classification accuracy when compared with the MFPINC method across the majority of datasets. It is noteworthy that the CPAT and CPPred methods did not reveal significant differences in classification accuracy within the *Gossypium darwinii* species dataset (z = 1.13, *P* = 0.23; z = 1.23, *P* = 0.29), respectively. Collectively, the statistical analysis indicates that there are indeed statistically significant differences between the MFPINC method and the methods ABLNCPP, CPC2, CPAT, CNIT, and CPPred.

**Table 4:** Results of significant differences between the MFPINC and the remaining six methods

Species	PINC		ABLNCPP		CPC2		CPAT		CNIT		CPPred	
	z-value	*p*-value	z-value	*p*-value	z-value	*p*-value	z-value	*p*-value	z-value	*p*-value	z-value	*p*-value
*Cicer arietinum*	1.13	0.22	1.40	0.16	3.00	0.01	2.50	0.01	4.80	0.003	2.88	0.003
*Gossypium darwinii*	3.59	0.0002	1.75	0.09	2.72	0.007	1.13	0.26	4.92	0.002	1.23	0.29
*Lactuca sativa*	1.45	0.17	1.55	0.11	4.75	0.00003	2.66	0.009	3.20	0.008	4.83	0.0001
*Manihot esculenta*	1.32	0.19	1.14	0.23	2.80	0.006	3.32	0.004	5.01	0.0005	5.76	< 0.00001
*Musa acuminata*	1.70	0.11	1.60	0.12	3.43	0.003	2.90	0.005	1.80	0.08	2.30	0.013
*Nymphaea colorata*	2.49	0.01	4.85	< 0.00001	3.00	0.004	3.53	0.002	4.95	0.0003	6.69	< 0.00001
*Sorghum bicolor*	1.10	0.27	1.80	0.09	3.10	0.003	1.01	0.30	4.70	0.0002	4.82	< 0.00001
*Zea mays*	0.48	0.59	1.00	0.30	3.79	0.001	3.20	0.002	1.75	0.08	3.10	0.005

## Materials and methods

**Fig. 10:**
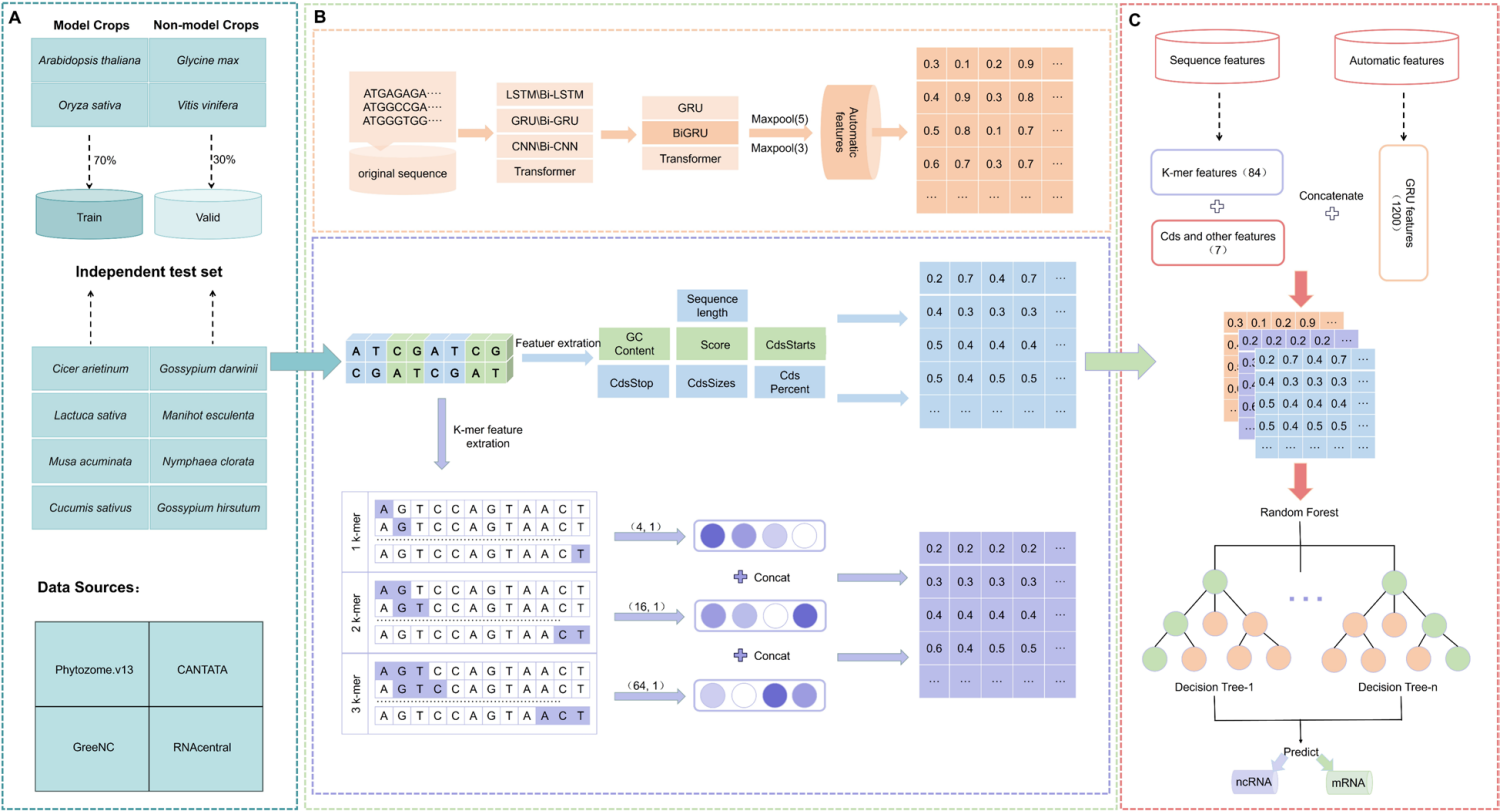
Flowchart of the development of our tool: (**A**) Dataset; (**B**) Feature acquisition processes; (**C**) Feature fusion and model construct

The development process of our method is shown below (Fig. 10), the model consists of two parts, 91 sequence features extracted manually in a random forest using a fusion of 1200 deep features extracted using the GRU model as input.

### Dataset construction

When constructing our experimental dataset, we meticulously considered two crucial factors: firstly, the biodiversity of plants and the abundance of annotated data, and secondly, ensuring data balance. To address these considerations, we selected four plant species as source materials for our training and validation datasets. This selection encompassed two model plants, *Arabidopsis thaliana,* and *Oryza sativa*, as well as two non-model plants, *Glycine max*and *Vitis vinifera*. Non-coding RNA (ncRNA) was utilized as positive samples, while coding RNA (mRNA) served as negative samples. The negative samples primarily originated from Phytozome.v13, whereas the positive samples were sourced from three widely utilized public databases: GreeNC [29], CANTATA [30], and RNAcentral [31].

During the data processing phase, we initially utilized the cd-hit-est-2D functionality within the CD-hit tool to eliminate redundant sequences with a similarity of 80% [32, 33] between the test and training sets [34]. Subsequently, to ensure dataset balance, we randomly selected samples for each plant type, resulting in a total of 4,000 samples per selection, comprising 2,000 positive and 2,000 negative samples. The table below provides a specific breakdown (Table 5), where the positive samples encompassed 1,800 long chain non-coding RNAs (lncRNAs) and 200 small molecule non-coding RNAs (sncRNAs), while the negative samples consisted of 2,000 mRNAs [35].

**Table 5:** Data setup for the training set of the model

Species	Noncoding		Coding	
	Total	Used	Total	Used
*Arabidopsis thaliana*	45,910	2000	27,416	2000
*Glycine max*	8599	2000	71,358	2000
*Oryza sativa*	11,338	2000	42,189	2000
*Vitis vinifera*	4301	2000	55,564	2000
Total	70,148	8000	196,527	8000

Hence, our benchmark dataset comprises a total of 16,000 gene sequences derived from four distinct plant species. Furthermore, we conducted an in-depth examination of the length distribution within both the positive and negative datasets. The median length of the coding RNA data is 1029, with a predominant concentration in the 0–2000 range. Conversely, the median length of ncRNA data is 321, primarily clustered within the 0–1000 range. Ultimately, we partitioned the dataset into two segments, allocating 70% for training data and 30% for validation data.

Furthermore, we generated eight distinct plant test sets (Table 6), namely *Cicer arietinum*, *Gossypium darwinii*, *Lactuca sativa*, *Manihot esculenta*, *Musa acuminata*, *Nymphaea colorata*, *Sorghum bicolor*, and *Zea mays*. To eliminate redundancy, the data for these eight independent test sets were sourced from the databases of the aforementioned four test sets and filtered using an 80% similarity threshold. Through this process, we successfully constructed an experimental dataset that not only encompasses an abundance of valuable information but also maintains a balanced distribution of data.

**Table 6:** Independent dataset

Species	Coding	Noncoding	Total
*Cicer arietinum*	2099	2099	4198
*Gossypium darwinii*	5622	5622	11,244
*Lactuca sativa*	4682	4682	9364
*Manihot esculenta*	2808	2808	5616
*Musa acuminata*	2059	2059	4122
*Nymphaea colorata*	1708	1708	3416
*Sorghum bicolor*	8657	8657	17,314
*Zea mays*	7406	7406	14,812

### Methodological overview

#### Choice of encoding method

Biological data often possesses a high degree of complexity and abstraction, while deep learning models demonstrate prowess in effectively tackling such intricacies, obviating the need for laborious manual feature engineering [36–40]. Traditional machine learning approaches have limitations in end-to-end learning of biological data, necessitating extensive understanding of biomolecules such as non-coding RNA (ncRNA) and coding RNA (mRNA). Conversely, deep learning models reduce the complexity of biological data analysis by automatically capturing informative features from the data itself, thereby mitigating the reliance on domain-specific knowledge and significantly simplifying biological data analysis. Moreover, deep learning empowers researchers with potent tools for handling high-dimensional datasets, enabling the automatic extraction of crucial features from raw, unprocessed sequence data. Capitalizing on these advantages, we intend to employ deep learning methods to investigate the profound distinctions between ncRNAs and mRNAs, aiming to achieve precise classification of these intricate biomolecules.

When conducting gene sequence prediction studies, it is crucial to consider how to retain the semantic information of the sequences and capture the diverse features inherent in gene sequences, including both similarities and differences, to enhance the accuracy of subsequent analyses [41, 42]. We employed one-hot and word-embedding encoding strategies throughout our experiments. By transforming discrete features into binary vectors, the one-hot encoding method encapsulates each base in a sequence, offering a precise representation of individual information fragments. Conversely, Word Embedding encoding seizes both the semantic and contextual information of the bases within a sequence, bestowing a richer understanding of the meaning, role, and interrelationships of the bases than what is offered by one-hot encoding. Recognizing the advantages of both one-hot encoding and word embedding encoding in these aspects, we initially conducted experiments to compare the predictive performance of seven deep learning models under these two encoding approaches [43, 44]. The results demonstrated that when utilizing word embedding encoding, the GRU [45], Bi-GRU, and Transformer models achieved comparable accuracies, surpassing the results obtained by the other models using the two encoding methods. Consequently, for our further research endeavors, we selected these three deep learning models that employ word embedding encoding to explore potential enhancements in gene sequence prediction.

#### Extraction of deep features

Considering that gene sequences typically exhibit highly intricate structures and sequence interdependencies, conventional feature extraction methods may struggle to capture these complexities effectively. Therefore, we opted to employ the aforementioned three deep learning models for extracting pertinent auto-generated features [28, 46–48], which could be utilized for further research.

**Fig. 11:**
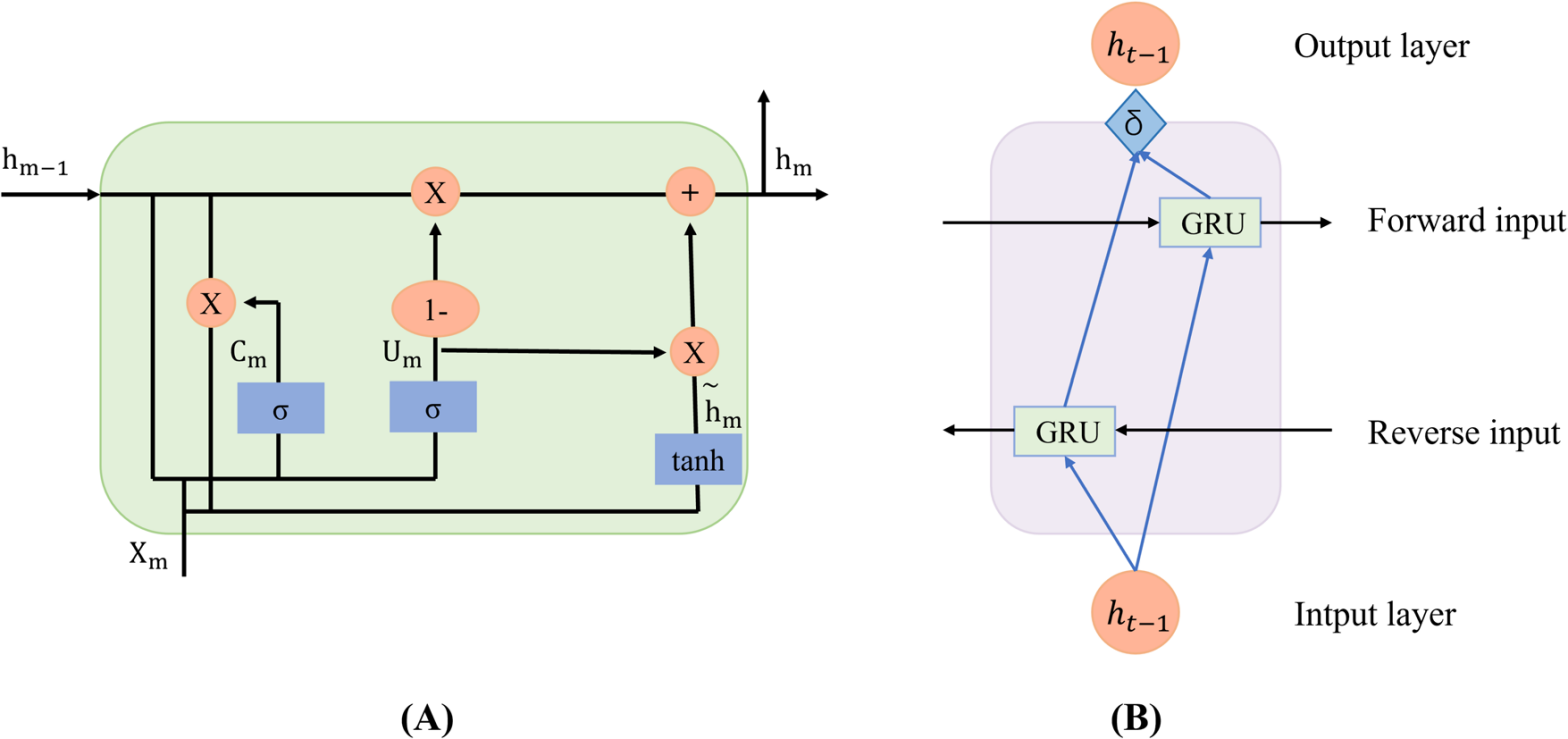
**A** GRU model; (**B**) Bi-GRU model

GRU is a variant of the RNN model that employs a reset gate and an update gate to determine how to discard and retain information. This approach effectively addresses the issue of long-term dependencies in data. Compared to the LSTM model, the GRU has fewer parameters and requires less computational effort. Despite these differences, the GRU can still achieve similar results to the LSTM. This makes the GRU a more efficient choice for training, as it can deliver comparable performance with less computational complexity. This paper uses the GRU model to obtain the context semantic information of text. Figure 11(A) depicts the GRU model’s structure.

The update gate is used to determine the impact of the previously hidden layer state on the current layer. The larger the value in the update gate, the greater the impact on the current layer at the previous moment. Formula (2) illustrates the update gate’s computation process.

2 $$U_m\;=\;\mathcal S(W_u\bullet\lbrack h_{\mathit m\mathit-\mathit1}\mathit,\mathit\;{\mathit\times}_{\mathit m}\rbrack)$$

The reset gate is used to remove the invalid information at the last moment. The smaller the reset gate value, the more invalid information is deleted. Formula (3) illustrates the reset gate’s computation process.

3 $$C_m\;=\;\mathcal S(W_c\;\bullet\lbrack h_{m-1},\;x_m\rbrack)$$

The current state computation method is presented in the following formulas:

4 $${h_m} = \tanh (W[{C_m} \odot {h_{m - 1}},{x_m}])$$

5 $${h_m} + (1 - {U_m}) \odot {h_{m - 1}}$$

Among them ${W}_{U}$, ${W}_{C}$ and ${W}$ represent the weight matrix of the GRU, ${x}_{m}$ is the input data, ${h}_{m}$ is the current hidden state of the model, the input of the previous state is ${h}_{m-1}$, ${\widetilde{h}}_{m}$ is the candidate active state, ${U}_{m}$ and ${C}_{m}$ represent update and reset gates, respectively, represents the Hadamard product, that is, the elements of the corresponding position are multiplied, and represents the sigmoid function.

Following our introduction of the foundational architecture of the GRU model, we proceed to delineate the structural intricacies of the Bi-GRU model. As shown in Fig. 11(B) above, Bi-GRU is a variant of recurrent neural networks that simultaneously captures the forward and backward dependencies of sequence data by combining two independent GRU layers, one of which processes the forward sequence and the other handles the reverse sequence. In the context of the Bi-GRU model, each sequence is subjected to forward and reverse inputs, which are then directed to the two distinct GRU layers. The forward GRU layer engages with the data by the sequence’s inherent order, whereas the reverse GRU layer addresses the data in the inverted sequence order. Consequently, the model is equipped to concurrently integrate both antecedent and subsequent information about each element within the sequence. Upon the computation of their respective hidden states by the two GRU layers, these states are consolidated to generate a unified representation that encapsulates the bidirectional contextual information of the sequence.

The Transformer model, as introduced by Google, is predominantly utilized in the domain of machine translation. Within the scope of this study, the Transformer is instantiated as an encoder-decoder framework. The architecture of the Transformer is characterized by the stacking of six layers within both the encoder and decoder components. Following the data’s traversal through the sequential layers of the encoder, it is subsequently relayed to the corresponding layer of the decoder to engage in the computation of attention mechanisms. The Transformer’s architectural composition encompasses four integral modules: an input module for data ingestion, an encoding module for sequence analysis, a decoding module for translating the encoded information, and an output module to generate the final translated sequence.

**Fig. 12:**
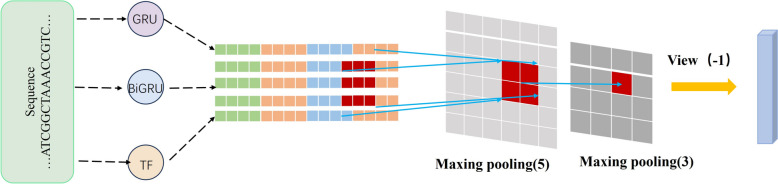
Deep feature extraction process

Upon the completion of selecting an appropriate feature extractor model, our initial step involved determining the optimal dimensionality for word embedding and defining the size of the hidden layers within the deep learning architecture. This process ensures that the preprocessed RNA sequences are efficiently transcribed into vectors of a predefined dimensionality, which are then subsequently inputted into our chosen deep learning model. As delineated in Fig. 12, for the GRU model, we identified an RNA sequence length of 1200 as the optimal input length. This model adeptly addresses the challenge of vanishing gradients and adeptly captures long-term dependencies within sequence data through the incorporation of update gates and reset gates. Concurrently, we implemented a maximum pooling layer with a kernel size of [5, 5] for feature extraction, which serves to downsample the time series while selectively preserving pivotal information. Subsequently, we integrated an additional max pooling layer with a [3, 3] kernel size, which further condenses the spatial dimensions of the features and amplifies their expressive power. Following these max pooling operations, we derived a set of automated features with a dimensionality of 1200. For the Bi-GRU model, we utilized an RNA sequence encoding of 1200 nucleotides in length as input for our experiments and employed two successive maximum pooling layers for feature extraction. By concurrently processing the time series in both forward and reverse dimensions, we procured a 1200-dimensional feature set that comprehensively encapsulates the contextual nuances of the sequence data. In the context of the Transformer model, we adopted a sequence encoding of 1000 nucleotides in length as input, harnessed the model’s self-attention mechanism to manage long-distance dependencies, and extracted features through two successive max-pooling layers, culminating in a 3000-dimensional set of automated features.

#### Extraction of sequence feature

To extract sequence features more conducive to integration with automatically generated features, we acquired three categories of sequence features in our experiments. These encompass k-mer frequency features, CDS-related features [49], and supplementary features (Table 7). K-mer represents a contiguous substring of k nucleotides (bases) within a gene sequence [50], employed for characterizing the local structure and composition of the gene sequence. K-mer frequency, conversely, signifies the occurrence count of each distinct k-mer nucleotide within the gene sequence. This feature vector furnishes us with a relative abundance of information regarding various k-mers within the sequence. For k = 1, each nucleotide can incorporate up to four distinct bases (A, G, C, T); for k = 2, there are a total of $[{4^2}$ = 16 associated 2-nucleotides (including AA, AG, … TC, TT, etc.); for k = 3, there are a total of $[{4^3}$ = 64 associated 3-nucleotides (including AAA, AAG, … TTC, TTT). Drawing insights from prior research, the amalgamation of 84 k-mer frequency features has been shown to enhance sensitivity in sequence alignment and similarity analysis [51], thereby facilitating improved detection of similarity and homology. Furthermore, varying levels of k-mer features enhance model flexibility, aiding in the discrimination of diverse categories of gene sequences. CDS features of gene sequences offer predictive insights into gene function, gene classification, gene expression, splice sites, and genetic variation. These predictive insights hold considerable significance for biological research, drug development, and precision medicine. These features encompass Score, cdsStarts, cdsStop, cdsSizes, and cdsPercent. Among these, Score represents a predictive score for a protein; when its value exceeds 800, there is a 90% likelihood that it signifies a protein, and if its value surpasses 1,000, it is almost certain to denote a protein. cdsStop denotes the terminal position of the coding region in the transcript, cdsSize reflects the length obtained by subtracting cdsStart from cdsStop, and cdsPercentage signifies the ratio of cdsSize to the total sequence length. Additionally, other features such as sequence length and GC content are extensively applied in ncRNA recognition. Sequence length denotes the total length of the sequence, while GC content represents the ratio of guanine and cytosine to the other four DNA bases.

**Table 7:** All features used in the text

Features	Description	Source
K-mer frequency	1–3 K-mer = 84 1 nucleotide = 4 features; 2 nucleotide = 16 features; 3 nucleotide = 64 features	MFPINC
Source	Values > 80 are likely to be a protein, And > 1000 must be a protein	TxCdsPredict
CdsStarts	Nucleotide position of CDS starts from the transcript And is based on zero	TxCdsPredict
CdsStop	Nucleotide position for the CDS end	TxCdsPredict
CdsSizes	CdsStop – CdsStart	TxCdsPredict
CdsPercent	(CdsStop + CdsStop)/total nucleotide sequence size	TxCdsPredict
Sequence length	Total nucleotide length of sequence	MFPINC
GC content	$\frac{C + G}{{A + C + G + T}}$	MFPINC

To mitigate the presence of redundant features among the initially extracted 91 features, we employed feature selection techniques [52, 53]. In our selection process, we initiated by applying a variance threshold to eliminate redundant features. Variance serves as a measure of data variability, thereby allowing us to assess the extent to which a particular feature exhibits variation. Features with extremely low variance indicate minimal fluctuations in the associated data, thereby severely limiting their potential to differentiate between distinct samples or contribute effectively to model training. By excluding features with a variance below the defined threshold, we not only diminished noise and redundant information but also enhanced the model’s ability to generalize well beyond the training data. Consequently, a total of 45 features remained after selecting the subset comprising features with variance higher than the mean. In addition to the variance thresholding approach, we employed a combination of variance thresholding filtering using the PINC tool and F-test to further filter redundant features. The F-test is a statistical method that allows us to assess the relationship between each feature and the corresponding label. By integrating these two approaches, we ultimately selected 10 features for subsequent analysis. The chosen features encompass GC content, Score, cdsStop, and cdsSize, as well as the frequencies of T, C, GC, GT, ACG, and TAT in the sequences.

#### Fusion of sequence features with deep features

**Fig. 13:**
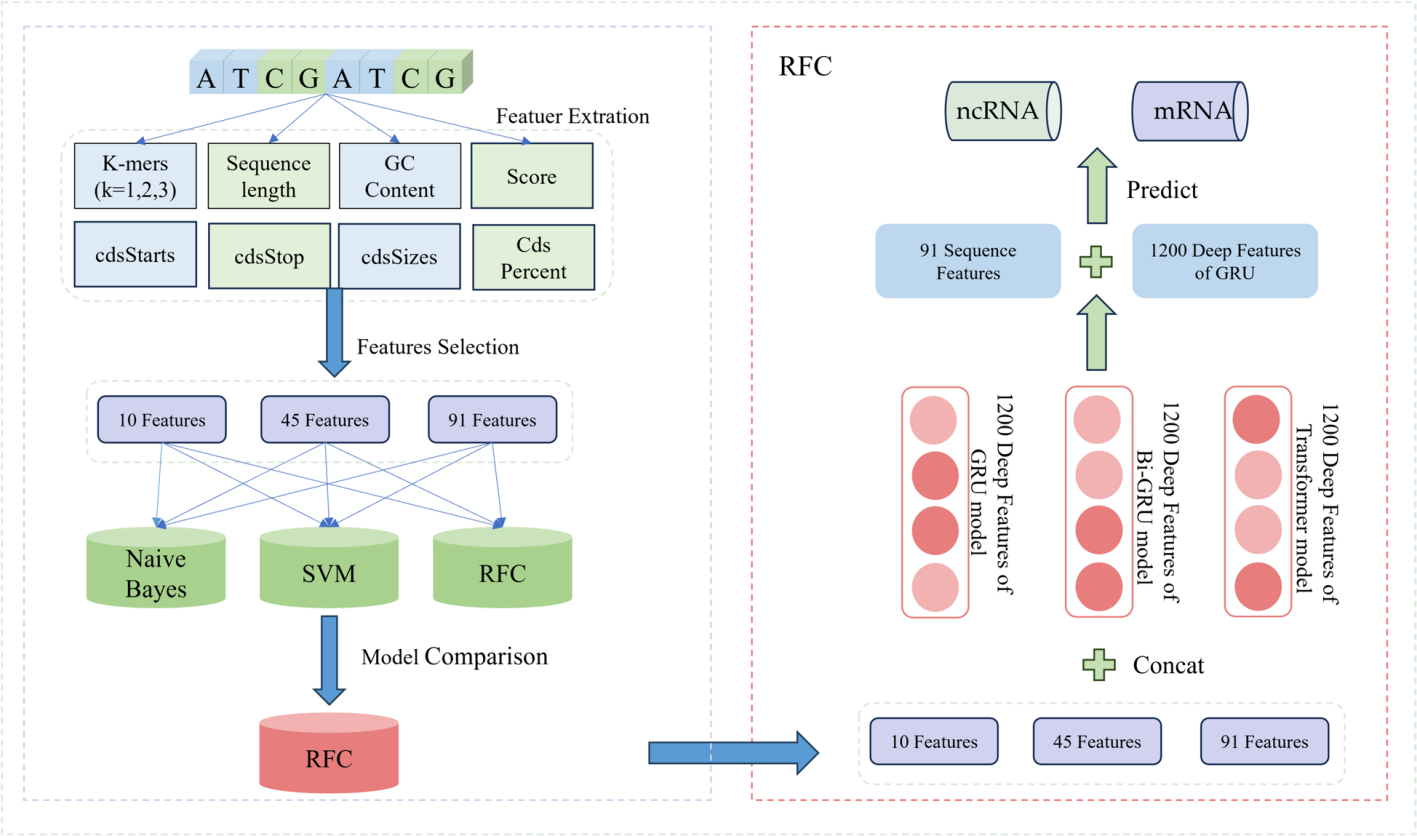
Detailed process of feature extraction and fusion

After extracting both sequence and depth features from the sequences and the deep model respectively, we proceeded with feature fusion and subsequent performance comparison (Fig. 13). We employed three different sets of sequence features – 10, 45, and 91 features – and integrated them with the deep features extracted from the GRU, Bi-GRU, and Transformer models. These depth features encompassed 1200 features from the GRU model, 1200 features from the Bi-GRU model, and 3000 features from the Transformer model. Following the fusion process, we utilized a training set comprising 16,000 sequences to train and predict the resulting nine fused features using three machine learning models: Naive Bayes, SVM, and RFC [41, 42]. Upon comparison, we observed that the RFC model yielded the most accurate predictions when all 91 sequence features were fused with the 1200 deep features extracted from the GRU model. Consequently, we adopted this approach, specifically fusing all 91 sequence features with the 1200 deep features extracted by the GRU model in the RFC model, for predicting the classification of gene sequences as non-coding RNA.

### Evaluation metrics

To evaluate our method, we used the following evaluation metrics namely Accuracy (ACC), Matthews Correlation Coefficient (MCC), Sensitivity (SE), Specificity (SPC), Positive Predictive Value (PPV), Negative Predictive Value (NPV), and F1 Score, which were used to measure the performance of our method and provide a comprehensive assessment of the prediction results. Their calculations are shown below:

$$Accuracy(ACC)=\frac{TP+TN}{TP+TN+FP+TN}$$

$$MCC\frac{TP\times TN-FP\times FN}{\sqrt{(TP+FN)\times(TP+FP)\times(TN+FP)\times(TN+FN)}}$$

$$Sensitivity(SE)=\frac{TP}{TP+FN}$$

$$Specificity(SPC)=\frac{TP}{TP+FN}$$

$$PPV=\frac{TP}{TN+FP}$$

$$NPV=\frac{TN}{TN+FN}$$

$$F1=\frac{2\times TP}{2\times TP+FP+FN}$$

where TP and TN indicate the number of correctly predicted ncRNAs and mRNAs and FP and FN indicate the number of incorrectly predicted ncRNAs and mRNAs. Precision indicates how many predicted ncRNA samples were correct. Recall indicates how many ncRNA samples were correctly predicted with a single class prediction accuracy for ncRNAs.The F1 score takes into account both the precision and the lookup rate, and its value is a coordinated average of them. The MCC, fully known as the Matthew correlation coefficient, integrates the TP, TN, FP, and FN, and can describe the prediction results correlation coefficient with the actual results. Its value ranges from -1 1, and the higher the value, the better the results of the model. By using these evaluation metrics, we were able to comprehensively assess the performance of our method in predicting cyclic RNA and obtain an assessment of the reliability of the prediction results.

## Discussion

Our method has demonstrated substantial advancements in the classification of ncRNAs. This result is mainly due to the effective combination of our multidimensional feature extraction techniques with advanced machine learning algorithms. When viewed through the lens of biological and biomedical research, our approach enhances not only the precision of ncRNA classification but also offers novel insights into the intricate biological functions of ncRNAs and their pivotal roles in cellular processes.

Within the domain of shallow feature extraction, we systematically derived k-mer features from ncRNA sequences, effectively capturing their local composition and sequence patterns. The process of extracting k-mer features is of significant biological importance because it can uncover conserved regions within the sequences that are frequently linked to the three-dimensional structure and functionality of RNA. Specific k-mer patterns can directly correspond to the binding sites for RNA-binding proteins, thereby influencing the stability and cellular function of ncRNAs. The identification of these locally conserved regions is crucial for understanding the role of ncRNAs in the regulation of gene expression, RNA editing, and other complex biological processes within the cell. Furthermore, the analysis of coding sequence (CDS)-related features significantly enhances our understanding of ncRNA functions. This enables us to identify ncRNAs that may be involved in coding-non-coding interactions, which are vital for comprehending the intricate regulatory network of gene expression.

The integration of the GRU model significantly enhances the process of deep feature extraction. This model is particularly adept at managing sequential data, discerning temporal dependencies, and identifying complex patterns within sequences. Consequently, it allows for the extraction of more nuanced and biologically significant features. These advanced features are crucial for improving the accuracy of classification tasks and provide a more profound understanding of the dynamic changes and cellular interactions associated with ncRNAs. Notably, the expression levels of specific ncRNAs may vary significantly during different stages of plant development or when subjected to environmental stress. Utilizing the GRU model, we can forecast these variations with greater accuracy, providing potential biomarkers for the investigation of plant stress response mechanisms. Furthermore, the GRU model is capable of simulating the dynamic expression patterns of ncRNAs throughout the plant cell cycle, shedding light on their roles in critical biological processes such as cell proliferation, differentiation, and programmed cell death. This approach affords a novel perspective for research in plant biology.

Our adoption of the RFC model has led to a significant enhancement in the precision of ncRNA classification and has opened new avenues for the identification of RNA functionalities. The efficacy of the RFC model is derived from the distinctiveness of each constituent decision tree. This distinctiveness arises from the strategy of random feature selection and bootstrap sampling, which imbues each tree with a measure of autonomy. The integration of fusion features provides a more comprehensive dataset for each decision tree, augmenting their variability, effectively reducing the overall model’s variance, and consequently, bolstering its generalization capabilities.By using multi-feature fusion technology, we can comprehensively analyze the role of ncRNA in various biological processes, including but not limited to plant cell differentiation, stress response, growth and development regulation, etc. This comprehensive analysis helps to deeply understand the functions and roles of ncRNAs in complex biological networks in plants, and provides new clues and research directions for plant stress biology and molecular breeding research.

Furthermore, our research methodology still holds potential for improvement. On one hand, we anticipate exploring and integrating higher-quality gene sequence features to enhance the performance of classification tasks and further optimize our models. On the other hand, a deeper investigation into the biological significance of different RNA types is necessary, including a more comprehensive understanding of their functions in cell biology and molecular biology. Such extensive research will contribute to unveiling the crucial role of RNAs in biological systems and provide essential guidance for future biotechnology research. In the realm of plants, numerous ncRNA functionalities remain unclear. Once these functions are identified, we will have the opportunity to explore novel biological mechanisms and integrate these newfound characteristics into our models, thereby further enhancing the efficacy of our tools. This field of study harbors immense research potential and holds the potential for significant breakthroughs in plant biology and agriculture. While our research methodology has already achieved substantial progress in the current phase, there is still ample room for future explorations. By continuously refining feature engineering techniques and deepening our comprehension of the biological aspects of RNA types, we are confident that we can further elevate the performance of our models and provide superior methods and tools for biotechnology research.

Our research method demonstrates excellent generalization ability, which makes the model more effective at adapting to new and unknown plant ncRNA data sets. In plant biology research, the identification of new types of ncRNAs often signifies the discovery of novel biological functions. Therefore, the advantages of our method in terms of generalization ability are of great scientific significance for the rapid identification of new ncRNAs in plants, the elucidation of plant stress responses, and the regulation of growth and development. An in-depth analysis of the ncRNA sequence is central to this study. Through detailed sequence analysis, we can identify and address the key difficulties and challenges in the classification process, and provide a clear direction for the enhancement of feature extraction and model optimization. This in-depth analysis will help us understand ncRNAs with special sequence characteristics and biological functions more comprehensively, and provide important clues for revealing their unique role in plant stress responses. Looking forward to the future, our method not only holds broad application prospects in the field of plant biology but also paves a new path for research in human health and medicine. For instance, by leveraging our research findings in the field of plant ncRNAs, we can better comprehend the function of ncRNAs in human diseases and foster the discovery and application of RNA markers in personalized medicine. These research results can not only deepen our understanding of the role of ncRNAs in the onset, progression, and treatment of diseases, but also offer innovative strategies and methods for early diagnosis, precise treatment, and individualized health management, thereby contributing to the advancement of human health.

## Conclusion

Currently, most tools used for ncRNA identification primarily rely on classification based on sequence and structural features. However, these features may not fully capture the functional and diverse nature of ncRNAs. To address this limitation, we propose a novel tool in our study that combines sequence features, deep features, and machine learning models to effectively differentiate between ncRNAs and mRNAs.

In our tool framework, we begin by utilizing the word embedding coding method to extract deep features from three different deep models. Additionally, we manually extract three sets of sequence features. Subsequently, we combine these different types of features and input them into a commonly used machine learning model for classification. To compare the effectiveness of different features, we analyze and contrast the deep features extracted from gene sequences in different depth models, the original sequence features, and the fused features. The results demonstrate that among the 91 features extracted, the fusion of 1200 deep features extracted using the GRU model achieves the highest prediction accuracy in the random forest model. To confirm the efficacy of our tool, we evaluate its performance on multiple datasets. The findings reveal that our tool exhibits superior prediction accuracy and robustness compared to existing tools. Moreover, our tool successfully facilitates ncRNA recognition across species of plants. To enhance usability for researchers, we also packaged the tools and placed them in Github (https://github.com/Zhenj-Nie/MFPINC) used for download and use, users can use detailed steps to predict and identify gene sequences.

In conclusion, our tool stands as a practical and user-friendly solution with excellent predictive performance and generalization capability. It represents a novel alternative for the identification and research of ncRNAs. Moving forward, we are committed to enhancing and refining the tool’s performance to meet the evolving demands of research. Furthermore, we aim to offer more valuable tools and resources for deeper exploration in the field of biotechnology.

## Data Availability

The datasets used and analyzed during the current research are available from the CANTATA database (http://rhesus.amu.edu.pl/CANTATA2/), GreeNC database (http://greenc.sequentiabiotech.com/wiki2/Main_Page), Phytozome.v13 database (https://phytozome-next.jgi.doe.gov/) and RNAcentral database (https://rnacentral.org/). The integrated data can be downloaded through the Github (https://github.com/Zhenj-Nie/MFPINC/tree/main/Data).
